# Prenatal screening for congenital anomalies: exploring midwives’ perceptions of counseling clients with religious backgrounds

**DOI:** 10.1186/1471-2393-14-237

**Published:** 2014-07-19

**Authors:** Janneke T Gitsels–van der Wal, Judith Manniën, Lisanne A Gitsels, Hans S Reinders, Pieternel S Verhoeven, Mohammed M Ghaly, Trudy Klomp, Eileen K Hutton

**Affiliations:** 1 Department of Midwifery Science, AVAG and the EMGO Institute for Health and Care Research, VU University Medical Center, Van der Boechorststraat 7, 1081 HV Amsterdam, Netherlands; 2 Faculty of Theology, VU University Amsterdam, De Boelelaan 1105, 1081 HV Amsterdam, Netherlands; 3 University College Roosevelt, Lange Noordstraat 1, 4331 CB Middelburg, Netherlands; 4 Center for Islamic Legislation & Ethics (CILE) Hamad Bin Khalifa University Qatar Foundation, P.O. Box 34110, Doha, Qatar; 5 Department of Midwifery Education Program, McMaster University, 50 Main Street East, Hamilton, Canada

**Keywords:** Counseling, Islam, Prenatal screening, Termination, Cultural competency, Shared decision-making, Congenital anomalies, Religion

## Abstract

**Background:**

In the Netherlands, prenatal screening follows an opting in system and comprises two non-invasive tests: the combined test to screen for trisomy 21 at 12 weeks of gestation and the fetal anomaly scan to detect structural anomalies at 20 weeks. Midwives counsel about prenatal screening tests for congenital anomalies and they are increasingly having to counsel women from religious backgrounds beyond their experience. This study assessed midwives’ perceptions and practices regarding taking client’s religious backgrounds into account during counseling. As Islam is the commonest non-western religion, we were particularly interested in midwives’ knowledge of whether pregnancy termination is allowed in Islam.

**Methods:**

This exploratory study is part of the DELIVER study, which evaluated primary care midwifery in the Netherlands between September 2009 and January 2011. A questionnaire was sent to all 108 midwives of the twenty practices participating in the study.

**Results:**

Of 98 respondents (response rate 92%), 68 (69%) said they took account of the client’s religion. The two main reasons for not doing so were that religion was considered irrelevant in the decision-making process and that it should be up to clients to initiate such discussions. Midwives’ own religious backgrounds were independent of whether they paid attention to the clients’ religious backgrounds. Eighty midwives (82%) said they did not counsel Muslim women differently from other women. Although midwives with relatively many Muslim clients had more knowledge of Islamic attitudes to terminating pregnancy in general than midwives with relatively fewer Muslim clients, the specific knowledge of termination regarding trisomy 21 and other congenital anomalies was limited in both groups.

**Conclusion:**

While many midwives took client’s religion into account, few knew much about Islamic beliefs on prenatal screening for congenital anomalies. Midwives identified a need for additional education. To meet the needs of the changing client population, counselors need more knowledge of religious opinions about the termination of pregnancy and the skills to approach religious issues with clients.

## Background

### Counseling on prenatal screening

Since 2007, prenatal screening of congenital anomalies has been offered to all pregnant women in the Netherlands, with more than 80% of pregnant women receiving counseling about prenatal screening from primary care midwives [1]. The number of counseling sessions offered by a fulltime midwife is on average 67 a year [2]. In the Netherlands, prenatal screening follows an opting in system and comprises two non-invasive tests: the combined test to screen for trisomy 21 (Down Syndrome) at 12 weeks of gestation and the second-trimester ultrasound examination to detect structural anomalies at 20 weeks. In cases of a positive screening followed by confirmatory diagnosis, two options are available: termination of the pregnancy before 24 weeks of gestation, or prenatal care focused on the health needs of the fetus and arrangements for postnatal support.

In the discussion on counseling about prenatal screening for congenital anomalies, it is generally accepted that the intended aim is to enable women who wish to be informed about the health of their future child to make an informed choice on the basis of the information they receive [3]. However, what constitutes ‘informed’ is not clearly specified [4]. Some of the literature on genetic counseling suggests that the counseling process should be ‘non-directive’ , and that counselors ought to abstain from making value judgments [5]. This may be interpreted to mean that counseling should be restricted to providing medical information about the anomalies tested for (such as trisomy 21), prenatal screening procedures and their risks, and the available options in the event of positive test results. Previous Dutch studies proposed going beyond ‘non-directive’ counseling, and introduced a shared decision-making model [6,7]. This approach of shared decision-making involves taking into account client’s personal standards and values [8]. Accordingly, Dutch midwives are trained according to a model that has taking the client’s perspective of life into account as its basis. This model, which is abbreviated as "MIMES", comprises Medical information, Individual choice by the client, Morally sensitive practices, Exploring the client’s values, and Supporting the decision-making [7,9]. Different roles are presented in this model; medical technical expert, advisor and teacher, and counselor in case of exploring values and supporting decision making. The MIMES model identified a number of factors that are relevant in test uptake decisions, including not only well-known factors such as age, parity, family life and personal experience, but also ‘identity markers’ such as ethnicity and religion [10-14]. It is acknowledged that the difficult questions that confront clients in decision-making may be influenced by their religious convictions [15-18]. As the shared decision-making approach prescribes taking the client’s perspective into account, the question arises as to whether asking about client’s religious convictions should also be part of the counseling process. A number of studies have answered this question affirmatively [19-23]. One study recommends a client-centered approach that includes concentrating on the cultural background of values, beliefs and behaviors of clients [19]. Other studies underline the role of religion on client decision-making, and suggest the importance of healthcare professionals having some knowledge of religious beliefs and convictions [20-23]. However, several studies have found that healthcare professionals do not possess this kind of knowledge [20,21,23-25].

### Islam and aspects of prenatal screening

Pregnant women with a Muslim background constitute a substantial part of the clientele of midwives in many western countries. In Islam, bioethical issues regarding decision-making on prenatal screening and termination of pregnancy have been worked out by for example the European Council for Fatwa and Research (ECFR) and the Islamic Organization for Medical Science (IOMS). Fatwas are statements, for instance by the above-mentioned organizations, that have authority and are respected by the average Muslim [26]. Worldwide, Islam has two main streams: Sunni (87-90%) and Shia (10-13%) [27]. Although differences of opinions exist between those streams, the basic principles of Islam are, for the most part, shared. There are small differences between and within both streams about aspects regarding prenatal anomaly screening such as beginning of life, moment of *ensoulment* and termination of pregnancy [28,29]. According to various Muslim scholars (both *Sunni* and *Shi’ite*), terminating pregnancy before the *ensoulment* is allowed when the life of the mother is in danger, or when the fetus has a serious anomaly [30]. Before the *ensoulment*, health of the mother could be interpreted in a broad sense as the physical and mental health, and social safety [30]. From the Islamic perspective, *ensoulment* is an important moment in pregnancy; from this moment on, the fetus is seen as a fully-fledged person. Based on the Prophetic traditions that can be read and interpreted in different ways, Muslim scholars have had different opinions about the timing of the *ensoulment*. The majority of Islamic theologians and legal experts agree that the *ensoulment* takes place on the 120^th^ day (17 weeks and one day) after conception. In terms of clinical practice, because gestation is considered to start two weeks earlier, the 120^th^ day translates to 19 weeks plus one day of gestation. A small minority of Islamic scholars believes it takes place on the 40^th^ day after conception (or seven weeks plus five days gestation in clinical practice) [28]. Termination after the *ensoulment* is seen as a crime against a human being, except when the life of the mother is at stake [28,30]. After the *ensoulment*, the life of the mother could be interpreted in a narrow sense as the physical and mental health. In 1990, Fatwa Number 4 by the Islamic jurisprudence council of Mekkah al Mukaramah officially confirmed the permissibility of termination of pregnancy in cases of serious anomalies before the 120^th^ day after conception [31-34]. This fatwa is also referred to by non-Muslim authors [17,28,30]. However, women’s reproductive choices regarding an affected child have not only been based on religious convictions; for example, the opinion of a medical expert about the severity of a fetal anomaly and life expectations may also influence women’s of couples’ reproductive choices [16,35]. An explanation of the flexible concept of a ‘serious’ anomaly is given by Rispler-Chaim: "the fetus is defective to a degree that it will never develop to live in a dignified normal life" [30]. Down’s syndrome, for example, ranges from mild to very serious forms and most women in our previous study among pregnant Muslim women from Turkish origin did not consider Down’s syndrome in general as severe enough to terminate a pregnancy [18].

In the same study, Muslim women who were interviewed said it would be helpful if midwives knew about the Islamic perspective on the meaning of life when giving prenatal counseling [18]. This view is supported by international literature showing that professional knowledge of religious and cultural backgrounds of their clients is important, but insufficient [8,33,36-38].

### Research questions

In this exploratory study, we investigated the extent to which Dutch midwives believe that they should take the religious background of their clients into account during prenatal counseling, whether they do so themselves and what factors play a role in whether religion is taken into account during counseling. We also explored the knowledge they possess on Islam’s position concerning termination, what factors influence this knowledge, and whether this knowledge differs between midwives who pay attention to the client’s religious background during counseling and those who do not.

## Methods

### Study design

This exploratory study is part of the DELIVER study. The DELIVER study is a multicenter prospective dynamic cohort study aimed at evaluating primary care midwifery in the Netherlands; its main focus is on quality, organization and accessibility of care. Between September 2009 and April 2011, data were collected from clients and midwives in twenty midwifery practices across the Netherlands by means of questionnaires, diaries with work-related activities, client records kept by the midwives and the linked data from the Netherlands Perinatal Registry. A complete overview of the design of the DELIVER study is given by Manniën *et al.*[39]. For this study, we used data from the DELIVER study questionnaires completed by clients and the questionnaires completed by midwives. The questionnaire was sent to 108 midwives by mail including a return envelope between March and June 2010. A reminder was sent to non-responders after four weeks. Privacy was guaranteed in accordance with Dutch legislation. Participants’ anonymity was maintained by using anonymous practice identifiers. All participants gave informed consent. The design and conduct of the study were approved by the Medical Ethics Committee of the VU University Medical Center Amsterdam.

### Participants

For the DELIVER study, twenty of the 519 primary care midwifery practices in the Netherlands were selected by means of purposive sampling, using strata that fulfilled three criteria. The first criterion was the region in which the practice is situated (north, middle or south of the country); the second criterion was the degree of urbanization (rural or urban area) and the third criterion was the practice type (dual or group practice). During the data collection, these practices employed a total of 108 midwives.

### Operationalization

The questionnaire for midwives contained questions about taking the religious background of pregnant women into account within the scope of counseling on prenatal screening (Table 1). Data were collected on midwives’ knowledge of preterm termination of pregnancy according to Islam by giving them four statements (one true and three false) with which they could agree or disagree. The statements with an indication of whether they are true or false in brackets were as follows:

– Termination is not allowed (false)

– Termination is allowed when the health of the mother is in danger (true)

– Termination is allowed when the child has Down syndrome (true)

– Termination is allowed when the child has severe congenital anomalies (true).

**Table 1:** Items from the questionnaire completed by midwives about counseling on prenatal screening

**Questions about taking the religious background of clients into account**	
1.	Do you think you should take the religious background of pregnant women and their partners into account during counseling? (yes/no)
2a.	Do you actually take the religious background of pregnant women and their partners into account during counseling? (yes/no)
2b.	If you do not take the religious background into account, what are the reasons why you do not do this? (multiple answers possible, as well as open-ended)
3a.	Do you counsel a pregnant Muslim woman differently to a pregnant non-Muslim woman? (yes/no)
3b.	If you counsel in a different way, in what way do you counsel Muslim and non-Muslim differently? (open ended)
**Questions about preterm termination of pregnancy with regard to Islam**	
1.	What do you know about termination of pregnancy with regard to Islam? (four statements: true/false)
2.	If a termination is allowed, until what gestational age is it permitted under Islam? (open ended or ‘don’t know’)
3.	If you do know anything about termination and Islam, what are your sources? (multiple answers possible, as well as open-ended)
4.	Do you need or are you interested in (more) education on religion with respect to prenatal screening? (yes/no)

We asked about the latest possible gestational age that termination of a pregnancy is allowed in Islam; where they obtained their knowledge; and whether they would be interested in more education about religion with regard to counseling on prenatal screening tests. Additionally, demographic data were collected, including age, gender, working experience as a midwife (number of years), and religion (Roman Catholic, Protestant, Muslim, Jewish, Buddhist, Hindu, humanist, none, did not know/would not say). In order to determine the proportion of the client population from various religious backgrounds at each of the twenty participating midwifery practices we used information from corresponding clients’ questionnaires that had been collected separately at each practice.

### Analysis

Information on the religious backgrounds of the clients in each practice was linked to the midwives’ questionnaire by an anonymous unique identifier. Descriptive statistics were used to summarize:

– Background characteristics of the midwives in the sample in comparison with all midwives in the Netherlands;

– The proportion of Muslims as well as religious clients per practice;

– The extent to which midwives took the religious background into account during counseling;

– Reasons for not taking the religious background into account;

– Reasons for counseling Muslim women differently to non-Muslim women;

– Knowledge about terminating pregnancy according to the Islam.

During preparatory analyses, dichotomous variables were constructed representing the religious background of the participating midwives (yes: Protestant, Catholic and Muslim versus no: none and humanist), low (<9%) versus high (≥9%) percentage of Muslim clients, interested in or in need of additional information (yes versus no and do not know).

The responses of midwives who claimed to pay attention to the clients’ religious background were compared to midwives who did not make that claim. Furthermore, the years of experience, age, midwife’s religious background (yes/no) and clients’ religious background (yes/no) of midwives who claimed to pay attention to the clients’ religious background were compared to midwives who did not make that claim.

A variable was constructed representing accuracy of knowledge about terminating pregnancy in Islam by using a sum score of correct answers to four statements, ranging from zero to four; the statements are presented above. This number of correct answers was compared across midwives with (or without) a religious background, across those who did and did not pay attention to the client’s religious background, and across practices with low and high percentages of Muslim clients. The independence between categorical variables was tested by means of *χ*^*2*^ tests. For comparisons across categorical or non-normally distributed variables, the non-parametric Mann–Whitney U test was used. The sizes of groups being compared could not be pre-specified, so statistical power is dependent on the variable used to define the subgroups. For all hypotheses, a significance level of 5% was chosen. SPSS (version 16) was used to analyze the data. Missing data for each variable of interest were pairwise deleted.

## Results

### Participants

The questionnaire was completed by 99 of the 108 midwives (response rate 92%). One questionnaire was excluded from the analyses because the midwife is the principal investigator, yielding a net response rate of 98 / 107 = 92%. At least one midwife per practice completed the questionnaire. Table 2 shows characteristics of the 98 midwives. The percentage of Muslim clients in the practices varied from 0% to 29.2%, median 3.4%. Three of the twenty practices had more than 9% Muslim clients, accounting for 18 of the 98 midwife respondents.

**Table 2:** Demographic characteristics of the study population (N = 98)

	**Sample of midwives**
	**N (%)**
**Age**	Median: 34.5 years; missing: 1
	<40 years: 56 (57%)
	>55% years: 6 (6%)
**Gender**	Male: 1 (1%)
	Female: 97 (99%)
**Experience**	≤ 5 years: 28 (29%)
	6-10 years: 20 (20%)
	≥ 11 years: 50 (51%)
**Religion**	Roman Catholic: 13 (16%)
	Protestant: 16 (20%)
	Muslim: 1 (1%)
	Humanist: 2 (2%)
	None: 47 (59%)
	Don’t know/would not say: 1 (1%)
	Missing: 18

### Accounting for religious background

Table 3 shows that 74 midwives (75%) stated that attention should be paid to the religious background during informing and counseling on prenatal screening, but 67 midwives (68%) reported actually paying attention to the religious background. The seven midwives with discordant responses mentioned two main reasons for not paying attention: firstly that they only pay attention when clients mention their religion; and secondly that religion slips their minds during counseling.

**Table 3:** Client’s religious background taken into account by midwives during counseling on prenatal screening

		**Actually pay attention**		
		**Yes**	**No**	**Total**
**Should pay attention**	Yes	67 (68%)	7 (7%)	74 (75%)
	No	1 (1%)	23 (24%)	24 (25%)
	Total	68 (69%)	30 (31%)	98 (100%)

Thirty midwives (31%) indicated that they did not actually pay attention to the religious background of the clients when counseling on prenatal screening. The main reasons as selected from pre-set multiple choice answers and open-ended answers were (Table 4): ‘religion is irrelevant in decision-making on prenatal screening’ (n = 13); the onus is on the client to bring religion into the discussion (n = 10); and ‘such discussion is not necessary because of the autonomous decision of the client’ (n = 7).

**Table 4:** Reasons of midwives for not taking the client’s religious background into account during prenatal counseling (N = 30)

**Reasons**	Number*
Survey-supplied reasons (closed)	
- Not enough time for counseling	0
- Religion is irrelevant	13
- It is stringent, difficult or stressful	0
- Not aware of this possibility	3
- Dutch privacy legislation prohibits data processing about religious backgrounds	2
Respondent-supplied reasons (open-ended)	
- The onus is on the client to bring religion into the discussion	10
- It is not necessary because of the autonomous decision of the client	7
- You have to approach every client as equal	3
- Only if the client hesitates about their decision	1
- Only in relation with the choice to terminate	1

*Respondents were allowed to select more than one option.

Eighty midwives (82%) said that they did not counsel Muslim women differently from women with other religious backgrounds (or none). Eighteen midwives (18%) indicated that they counsel Muslim women differently. Four said they do so because of cultural and language differences. Eight midwives indicated that they counsel Muslim women in a different way, stating that they explain that choosing to have prenatal screening tests does not necessarily mean choosing termination if the result is unfavorable (n = 2); that they start the counseling with the possibility of termination (n = 4); or that they take the religion into account at the beginning of the counseling (n = 2). One midwife told women who would not consider terminating the pregnancy that the screening tests are not useful.

The percentage of clients with a religious background did not vary significantly between midwives who did or did not pay attention to the religious background during counseling (*Mdn* = 40.8 and 35.8 respectively, *p =* 0.54), nor was paying or not paying attention related to the length of the counselors’ experience (*Mdn =* 9 and 14 years respectively, *p =* 0.19).

Furthermore, midwives’ own religious backgrounds were independent of whether they paid attention to the clients’ religious backgrounds (*χ*^*2*^ (1) 0.85, *p =* 0.36). Midwives who did not pay attention to the client’s religious background were older (*Mdn =* 42) than those who did pay attention (*Mdn =* 33; *p =* 0.07); there was not enough statistical power to interpret this outcome as significant. An analysis by gender was not performed, because there was only one male midwife.

### Knowledge of termination allowance in Islam

Midwives’ accuracy of knowledge on the Islamic perspective on termination is shown in Table 5. Seventeen participants thought that Islam did not allow termination under any condition. Two participants (2%) answered all four statements correctly and 25 participants (25%) answered none of the statements correctly; the median of correct answers given is one.

**Table 5:** The number (percentage) of participants who correctly answered the statements about permissibility of termination from an Islamic perspective

**Statements**	**Midwives with <9% Muslim clients (N = 88)**	**Midwives with ≥9% Muslim clients (N = 18)**	**Total (N = 98)**
	**Correctly answered**	**Correctly answered**	
	**N (%)**	**N (%)**	
Termination is not allowed (false)	56 (70)	15 (83)	71 (72)
Termination is allowed when the health of the mother is in danger (true)	22 (28)	13 (72)	35 (36)
Termination is allowed when the child has Down syndrome (true)	2 (3)	1 (6)	3 (3)
Termination is allowed when the child has severe congenital abnormalities (true)	11 (14)	1 (6)	12 (12)

Eighty-one midwives (83%) indicated that they have no knowledge about the latest possible gestational age to terminate a pregnancy with regard to Islam. Of the other seventeen participants (17%), none gave the correct answer of 40 or 120 days after conception, that is 9 weeks plus one day or 19 weeks plus one day of gestation. The given answers ranged from 6 to 24 weeks of gestation. Midwives’ knowledge about Islamic attitudes to termination came from diverse sources, such as pregnant women (n = 7), a colleague (n = 4) or a course (n = 1); one midwife is Muslim herself. None of the midwives answered ‘media’ or ‘midwifery education’ , which were available options. Sixty-five per cent of the responding midwives were interested in additional education about Islamic religious beliefs with respect to counseling on prenatal screening tests of congenital anomalies. Twenty percent of the midwives were not interested in additional education and 14% had no opinion.

Midwives from practices with a lower percentage of Muslim clients had fewer correct answers regarding Muslim beliefs compared to midwives whose practices cared for a higher proportion of Muslim clients (*Mdn =* 1.0 and 2.0 respectively, *p =* 0.02). There was no difference in the number of correct answers between midwives with a religious background (*Mdn =* 1) and midwives without a religious background themselves (*Mdn =* 1, *p =* 0.45). No difference in the number of correct answers was found between the midwives who paid attention to the clients’ religious background (*Mdn =* 1) when counseling on prenatal screening tests and midwives who did not (*Mdn =* 1, *p =* 0.82).

## Discussion

Although several studies emphasize the importance of considering religious convictions in decision-making on prenatal screening [15,17], our exploratory study of Dutch midwives shows that a substantial number (31%) do not pay attention to the religious background of their clients. Reasons for not paying attention to the client’s religious background can be categorized into normative and non-normative reasons. Some of the latter are indicated by an American study arguing that healthcare professionals lack enough time to discuss these matters, and often feel uncomfortable doing so [20]. It should be noted that none of the midwives in our study mentioned these non-normative reasons for not including clients’ religious convictions as part of their prenatal counseling. Lack of time might not be an issue for Dutch midwives, as prenatal screening has been included in the standard prenatal care in the Netherlands and midwives can add 15 billable minutes to the time available for each client. The same American study confirmed our findings that healthcare professionals’ own religious background or their length of experience were not factors that influenced whether they discuss religion with their clients [20]. In the Dutch situation, the above factors can be expected to be comparable. The introduction of counseling for prenatal screening in midwifery practice was only three years ago, and all midwives received the same training in the MIMES model, teaching them the importance of the client’s values and beliefs in decision-making on prenatal screening. Regardless of their own convictions, midwives were taught that addressing their clients’ religion is part of what is involved in the shared-decision making approach. The number of years of experience does not therefore genuinely reflect midwives’ counseling experience.

In our study, midwives who did not pay attention to the client’s religious background did so for normative reasons. Some midwives rejected paying attention to religion by underlining the client’s autonomous decision-making; some respondents felt that religious convictions are irrelevant in prenatal screening decision-making; and others stated that they only pay attention to religion when clients themselves bring it up. These normative reasons for not taking religion into account in prenatal counseling fall on different points along a spectrum of non-directive approaches to counseling; with abstention from discussion at one end (a stronger claim) and restricting discussion of religion to the client’s initiative at the other (a weaker claim).

Rejecting the stronger claim, we argue for the value of a client-centered and shared decision-making approach for the clients. This approach involves providing relevant information and discussing this information from the perspective of the client’s personal values and beliefs. In this context, it can be suggested that sharing and discussing the client’s own perspective is actually enhancing their autonomy, as has been argued persuasively from the perspective of care ethics [40,41]. The argument for rejecting the weaker claim is ultimately the same. While this view is compatible with recognizing the importance of discussing the client’s religious background, it fails to acknowledge the nature of a *shared* decision-making process, and advocates a passive role for the care provider. Both approaches can thus be seen to be incompatible with shared decision-making; the approach that is endorsed by the midwifery prenatal screening program and is integrated in the MIMES training program.

The next point is that our exploratory study indicated that the participants had little knowledge about the termination of pregnancy according to Islam in the case of congenital anomalies. Although the midwives with a higher percentage of Muslim clients (more than 9%) had more knowledge of Islamic attitudes to terminating pregnancy in general than the midwives with a lower percentage of Muslim clients, the specific knowledge in both groups of termination with regard to trisomy 21 and other congenital anomalies was limited. No midwives in our study knew the latest day of legitimate termination as taught by Islamic sources. Starting from the first day of the last menstrual period, the last day of legitimate termination is nineteen weeks plus one day of gestation, which is one week before the second-trimester ultrasound is offered in the Netherlands. This means that, for a Muslim woman living in the Netherlands who is guided by her religious beliefs regarding the moment of ensoulment (120 days after conception) information indicating a serious anomaly resulting from routine second-trimester ultrasound screening will be received too late to inform her choice regarding continuation of the pregnancy. Similarly, previous studies showed that not all pregnant Muslim women themselves have sufficient knowledge of the rulings of their own specific tradition on termination in the case of a congenital anomaly [18,35]. Neter *et al.* observed similar findings and argued that midwives, in their role as counselors, are expected to inform Muslim women about prenatal screening tests and to discuss the possibility of termination if it comes to a fetus with serious anomalies [37]. In an Egyptian study, the fatwa that permits termination in case of a serious anomaly up to 120 days after conception was discussed with couples at risk of fetuses with thalassemia. After these in-depth counseling sessions, all mothers with confirmed thalassemia fetuses opted to terminate their pregnancies [42]. This suggests that, within their system of beliefs, the mothers may opt for termination. The latter two studies underline the importance of taking clients’ religious backgrounds into account in order to enhance client autonomy as part of a shared decision-making model. Many researchers believe that a prerequisite for counseling on prenatal screening is sufficient knowledge of the cultural and/or religious background of the client’s values and beliefs [23,25,33,36-38]; this is particularly important when termination of pregnancy is considered [36,37]. A qualitative Canadian study found negative experiences among immigrant Muslim women as a result of insensitivity and lack of knowledge on the part of their prenatal care providers about their religious and cultural background [25]. Hasnain *et al.* investigated provision of culturally appropriate and client-centered care to Muslim women in the US and they recommended education for healthcare professionals focused on basic religious and cultural beliefs of Muslim women [23]. Furthermore, our study also found that the majority of counselors (65%) needed additional education about religious beliefs with respect to counseling on prenatal screening tests for congenital anomalies. This education is required for a meaningful implementation of the shared decision-making approach.

As far as we are aware, this is the first study to ask midwives if they pay attention to the client’s religious background in their role as prenatal counselors. This study is also the first to explore counselors’ knowledge about termination from a specific religious (Islamic) perspective. A particular strength of the study is the high response rate of 92%; however, the external validity may be questioned because of the selected sampling of midwifery practices that were included, and the reliability may be questioned because of the small overall numbers. In particular the results of the subgroups analyses include very small numbers and limit the generalizability of these findings.

There are some recommendations for further research. In our exploratory study, midwives reported on their own behavior for taking account of the client’s religious background. The question is whether their reports would be confirmed by other, more objective, research methods like video-taped counseling sessions. Further research with a larger sample size and unselected sampling will be important to enhance the generalizability of the results. A client-centered and shared decision-making approach to prenatal counseling warrants additional research that investigates the views and experiences of pregnant women and their partners. Finally, additional research may be warranted to assess what is to be considered as sufficient knowledge about religious aspects and how this should be included in professional education and training.

## Conclusion

One-third of the midwives did not pay attention to the religious background when informing and counseling clients about prenatal screening tests for congenital anomalies. Furthermore, midwives had limited knowledge of termination according to Islamic beliefs and identified a need for additional training. In order to meet the needs of the changing client population, midwives in the context of the broader healthcare system need more detailed knowledge about religious beliefs. At the care provider level, more attention should be paid to the religious background of clients during counseling in order to improve shared-decision making on prenatal screening for congenital anomalies. At the systems level, within the Netherlands and in the case where the fetus does have a serious congenital anomaly, women from the largest non-western religion (i.e. Islam) have limited access to the option of terminating the pregnancy, because of the timing of the second-trimester ultrasound. Revision of the timing of this ultrasound examination should be considered.

## Abbreviations

MIMES: Medical information, Individual choice by the client, Morally sensitive practices, Exploring the client’s values, Supporting the decision-making.

## Competing interests

The authors declare that they have no competing interests.

## Authors’ contributions

JG, JM, HR and MG developed the questionnaire. JG and JM conducted the coding of open answered questions and JG, LG, and NV conducted the statistical analyses. TK initiated and coordinated the DELIVER study. EH supervised the DELIVER study and the current study. JG drafted the manuscript, and all authors read and corrected draft versions of the manuscript and approved the final manuscript.

## Pre-publication history

The pre-publication history for this paper can be accessed here:

http://www.biomedcentral.com/1471-2393/14/237/prepub
